# Operating room organization and surgical performance: a systematic review

**DOI:** 10.1186/s13037-023-00388-3

**Published:** 2024-01-29

**Authors:** Arnaud Pasquer, Simon Ducarroz, Jean Christophe Lifante, Sarah Skinner, Gilles Poncet, Antoine Duclos

**Affiliations:** 1 Research On Healthcare Performance RESHAPE, Université Claude Bernard, Inserm U1290, Lyon 1, France; 2 Department of Digestive and Colorectal Surgery, Edouard Herriot University Hospital, 5 Place d’ Arsonval, 69003 Lyon, France; 3 Health Data Department, Hospices Civils de Lyon, France; 4 INSERM, UMR 1052-UMR5286, UMR 1032 Lyon Cancer Research Center, Faculté Laennec, Lyon, France; 5 Lyon University, Claude Bernard Lyon 1 University, Villeurbanne, France; 6 Department of Endocrine Surgery, Hospices Civils de Lyon, Lyon, France

**Keywords:** Surgery, Staffing, Turn over, Familiarity, Teamwork, Disturbing elements, Scheduling, Workload

## Abstract

**Background:**

Organizational factors may influence surgical outcomes, regardless of extensively studied factors such as patient preoperative risk and surgical complexity. This study was designed to explore how operating room organization determines surgical performance and to identify gaps in the literature that necessitate further investigation.

**Methods:**

We conducted a systematic review according to PRISMA guidelines to identify original studies in Pubmed and Scopus from January 1, 2000 to December 31, 2019. Studies evaluating the association between five determinants (team composition, stability, teamwork, work scheduling, disturbing elements) and three outcomes (operative time, patient safety, costs) were included. Methodology was assessed based on criteria such as multicentric investigation, accurate population description, and study design.

**Results:**

Out of 2625 studies, 76 met inclusion criteria. Of these, 34 (44.7%) investigated surgical team composition, 15 (19.7%) team stability, 11 (14.5%) teamwork, 9 (11.8%) scheduling, and 7 (9.2%) examined the occurrence of disturbing elements in the operating room. The participation of surgical residents appeared to impact patient outcomes. Employing specialized and stable teams in dedicated operating rooms showed improvements in outcomes. Optimization of teamwork reduced operative time, while poor teamwork increased morbidity and costs. Disturbances and communication failures in the operating room negatively affected operative time and surgical safety.

**Conclusion:**

While limited, existing scientific evidence suggests that operating room staffing and environment significantly influences patient outcomes. Prioritizing further research on these organizational drivers is key to enhancing surgical performance.

**Supplementary Information:**

The online version contains supplementary material available at 10.1186/s13037-023-00388-3.

## Introduction

The success of a surgical procedure is not solely determined by the specific surgical intervention itself or patient-related factors, but rather relies on the comprehensive quality of care provided to the patient during their hospital stay [1]. This encompasses the combined efforts of numerous healthcare professionals involved in the patient's treatment, whose individual performances are intricately influenced by the environment in which they operate [2]. Therefore, the outcome of surgery appears multifaceted and could be related to the collaborative synergy and environmental factors that impact the overall delivery of care [1, 2]. While risk factor identification for surgical complications has traditionally focused on patient comorbidities and the surgical procedure itself, postoperative complications may also depend on the organization of the operating room. Previous investigations have highlighted the significance of team interaction and team learning curves in this context [3–5]. Other studies have examined determinants such as teamwork, measured using teamwork assessment scales, and intraoperative failures [6, 7], team communication [8, 9], resident participation [1, 10–12], music listening [13–15], task interruptions [16], and organizational parameters [17]. However, those studies to date are based on qualitative approaches with narrow scope, focusing on specific procedures or outcomes.

A comprehensive understanding of the relationship between organizational factors and surgical outcomes remains elusive due to the absence of syntheses in this broad and heterogeneous field. Existing reviews have not adequately covered the range of determinants and outcomes beyond the patient and the surgical procedure, and they are often descriptive or focused on only one determinant, without any general overview of the complex interactions that can occur between the determinants. To address this lack of synthesis of this broad field and to identify research gaps, we conducted a systematic review, based on available quantitative studies, to explore the influence of organizational factors in the operating room on surgical performance and patient outcomes.

## Methods

### Search strategy

A preliminary search was conducted to identify articles that aligned with the research theme and develop a comprehensive search strategy. This preliminary search lead to determine five major organizational factors categories and three main clinically significant surgical outcomes, as follows: 1) Team composition, 2) Team stability, 3) Team work, 4) Work scheduling, 5) Disturbing elements. Surgical outcomes were categorized as follows: 1) Operative time, 2) Surgical safety, 3) Economic resource consumption. The databases used for the study included PubMed and Scopus, and the search algorithm was adapted for each database. The full research algorithms used for each database are outlined in Additional file 1: Appendix I. The reference list of included articles and any relevant systematic reviews were also checked for additional studies. Studies published in English from January 1st, 2000 were considered for inclusion. Eligible studies included those from any geographical location, that involved professional surgeons or surgical trainees (such as fellows or residents), regardless of their specialty. Only quantitative studies based on original research investigation were considered while qualitative studies were disregarded, as well as systematic reviews, comments, and opinion papers. Both observational (cross-sectional and longitudinal designs) and interventional (quasi-experimental designs and randomized experimental designs) studies were considered for inclusion. The included studies focused on measuring and assessing the association between organizational factors in the operating room and surgical performance. Only studies based on real surgical procedures performed inside the operating room were considered, as opposed to simulated interventions or simulation training conducted outside the operating room. Studies were screened according to the five determinants and three identified outcomes. All identified citations were collated and uploaded into Endnote bibliographic software and duplicates were removed. Titles and abstracts were screened for selection by two independent reviewers (AP and SD) for assessment against the review’s inclusion criteria. The full texts of selected citations were then retrieved and assessed in detail against the inclusion criteria by the same two independent reviewers. Reasons for exclusion of sources of evidence at full text that did not meet the inclusion criteria were recorded and reported. Any discrepancy between the two reviewers during the selection process was resolved through agreement or with an additional reviewer (AD) if no consensus was found. The results of the citation screening and the study inclusion process was fully reported in the final systematic review and presented in a Preferred Reporting Items for Systematic Reviews and Meta-analyses extension for scoping review (PRISMA- ScR) flow diagram [18].

### Data extraction

Data were systematically collected from studies included in the systematic review using a previously developed extraction tool (see Additional file 2: Appendix II). The data extracted included specific details about the study methods and findings relevant to the review questions (see Additional file 2: Appendix II). As definitions of organizational factors are different from one publication to another, we grouped and categorized organizational factors investigated in selected studies into five categories, as follows: 1) team composition: number of participants (surgeons, residents, anesthetists, nurses) during surgery, level of experience of participants, and surgical team relationship (supervised work, involvement of residents, surgeon/resident-nurse-anesthetist relations). 2) team stability: number of former collaborations (surgeon/resident, surgeon/anesthetist, surgeon/nurse), turnover of the surgical and anesthetic team between procedures or during a same procedure. 3) team work: scales measuring teamwork, leadership, communication inside of the team, including communication failure. Teamwork in the operating room refers to the coordinated and collaborative efforts of multidisciplinary healthcare professionals working together seamlessly to achieve optimal patient outcomes 4) work scheduling: patient order, or modifications in the scheduling of surgical procedures, dedicated operating rooms, patient turn over, work overlay. 5) disturbing elements: number of disturbing elements during surgical the procedure, type or duration of disturbance.

Surgical outcomes were categorized as follows: 1) operative time, 2) surgical safety (i.e. morbidity, mortality, redo surgery, readmission), 3) economic resource consumption (i.e. cost and length of stay).

### Data analysis

The quality of the studies was assessed in a standardized manner by assigning a quality score based on the presence or absence of three methodological criteria: detailed description of the size of the patient population and the number of participating healthcare professionals, multicenter (i.e. more than one center or hospital) study setting, and longitudinal or randomized study design. One point was attributed for each criterion present.

Data analysis involved a review and classification of various aspects of the organizational factors, including the study setting, objectives, outcomes, determining factors, study design, statistics, and results. For quantitative variables, when the outcome was found in several studies, the median value was calculated. When statistical analysis was carried out in the included manuscripts and multiple results were obtained, the median value was calculated. The statistically significant results were used to differentiate between positive and negative studies. If statistical analysis was not performed or not significant, the results were classified in the section for neutral studies. The data was presented graphically when appropriate, following appropriate guidelines for systematic reviews [19].

This systematic review was carried out in accordance with PRISMA guidelines and the methodology for scoping reviews further outlined by Arkset and O’Malley [20–22]. The Protocol was registered on the Open Science Framework (OSF) with the following: 10.17605/OSF.IO/WBF9S.

## Results

**Fig. 1:**
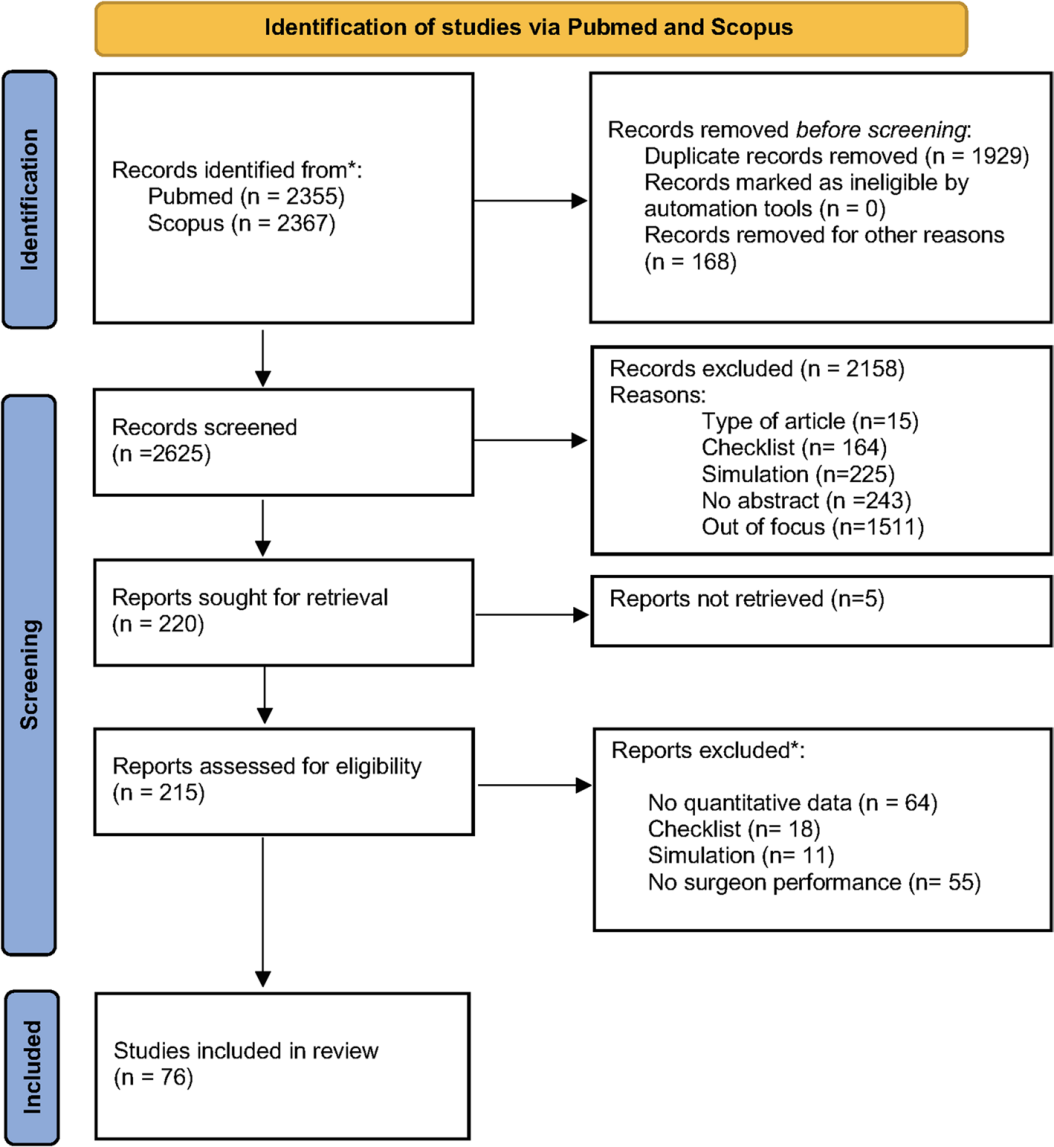
PRISMA flowchart

Of 2625 identified references, 220 abstracts were deemed potentially suitable. After a thorough evaluation of the full text, 76 articles were selected. According to our search strategy, the inter-rater agreement kappa was 0.78 for title and abstract screening; and 0.87 for full texts screening. The PRISMA-ScR flow chart of the systematic review is depicted in Fig. 1. The most researched specialties (Table 1) were digestive and general surgery (39.4%) and orthopedics (17.1%). Most studies were conducted at a single center in North America (69.7%) and mainly focused on elective surgeries (77.1%). The number of procedures analyzed ranged from 6 to 89,720 (median = 1031). The majority of study designs were observational (88.1%) rather than interventional (11.7%).

**Table 1:** Characteristics of populations and studies

Overall number of studies	*N* = 76
**Geographical area of the study or of the author’s affiliations**	*N (%)*
North America	53 (69.7)
Europe	20 (26.3)
Asia	3 (4)
**Number of centers**	*N (%)*
Monocentric	46 (60.5)
Multicenter *[median 4.5 (2–258)]*	30 (39.5)
**Surgical specialty**	*N (%)*
Digestive and general	30 (39.4)
Orthopedic	13 (17.1)
Cardiovascular and thoracic	9 (11.8)
Pediatric	9 (11.8)
Urology	7 (9.2)
Gynecology	6 (7.8)
Neurosurgery	4 (5.2)
Ophthalmology and Otorhinolaryngology	3 (4)
Undifferentiated	1 (1.3)
**Operating room team**	*Median (range)*
Number of surgeons *(n* = *52)*	3 (1–688)
Number of surgical resident *(n* = *33)*	11 (1–1396)
Number of anesthetist *(n* = *7)*	15 (1–168)
Number of nurses* (n* = *10)*	72 (7–3432)
**Population**	*Median (range)*
Number of patients operated *(n* = *72)*	1031 (6–89,720)
**Type of surgery evaluated**	*N (%)*
Laparoscopic/endoscopic/endovascular	29 (38.1)
Open	27 (35.5)
Robotic	4 (5.2)
Missing	30 (39.4)
**Scheduled/emergency procedure**	*N (%)*
Elective	59 (77.1)
Emergency	3 (4)
Missing	14 (18.9)
**Study Design**	*N (%)*
Observational study	67 (88.1)
Cross-sectional	46 (60.5)
Longitudinal	21 (27.6)
Interventional study	9 (11.7)
Quasi-experimental study	5 (6.5)
Randomized trial	4 (5.2)
**Data source**	*N (%)*
Ad hoc register	22 (28.9)
Medico-administrative	11 (14.4)
Electronic health records	60 (78.9)

**Fig. 2:**
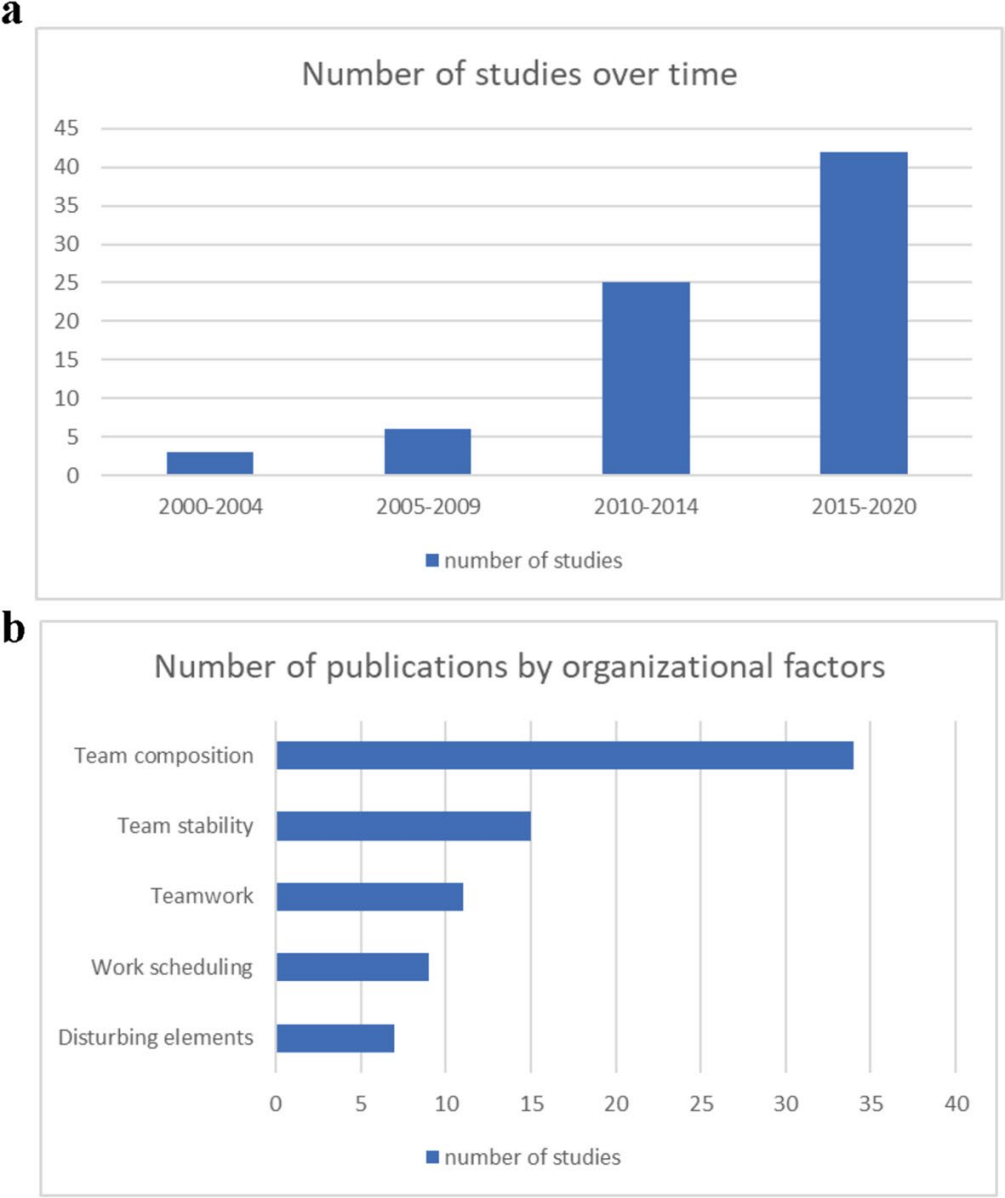
**a **Number of studies according to time.** b** Number of publications by organizational factor category. Legend: Definitions of organizational factors: Team composition = number and experience of surgeon, residents, anesthetists, nurses; surgical team relations (work under supervision, involvement of residents, surgeon/resident-nurse-anesthetists relations. Team stability = number of former collaborations (surgeon/resident, surgeon/anesthetist, surgeon/nurse), turnover of surgical and anesthetic team between procedure or during a same procedure. Team work = measuring scales of teamwork, leadership, communication inside team including communication failure. Disturbing elements = number of disturbing elements, type of disturbance, duration of disturbance. Work scheduling = order of scheduling, modifications in scheduling, dedicated operating room, patient turn over, work overlay

The investigation of organizational factors has become a growing theme over time with 89% (68/76) of studies published after 2010 (Fig. 2a). Overall, 34 (44.7%) studies investigated the role of the surgical team composition, 15 (19.7%) looked into the effect of team stability, 11 (14.5%) examined the effect of team work, 9 (11.8%) studied the influence of work scheduling and 7 (9.2%) explored the effects of disturbing elements in the operating room (Fig. 2b).

**Fig. 3:**
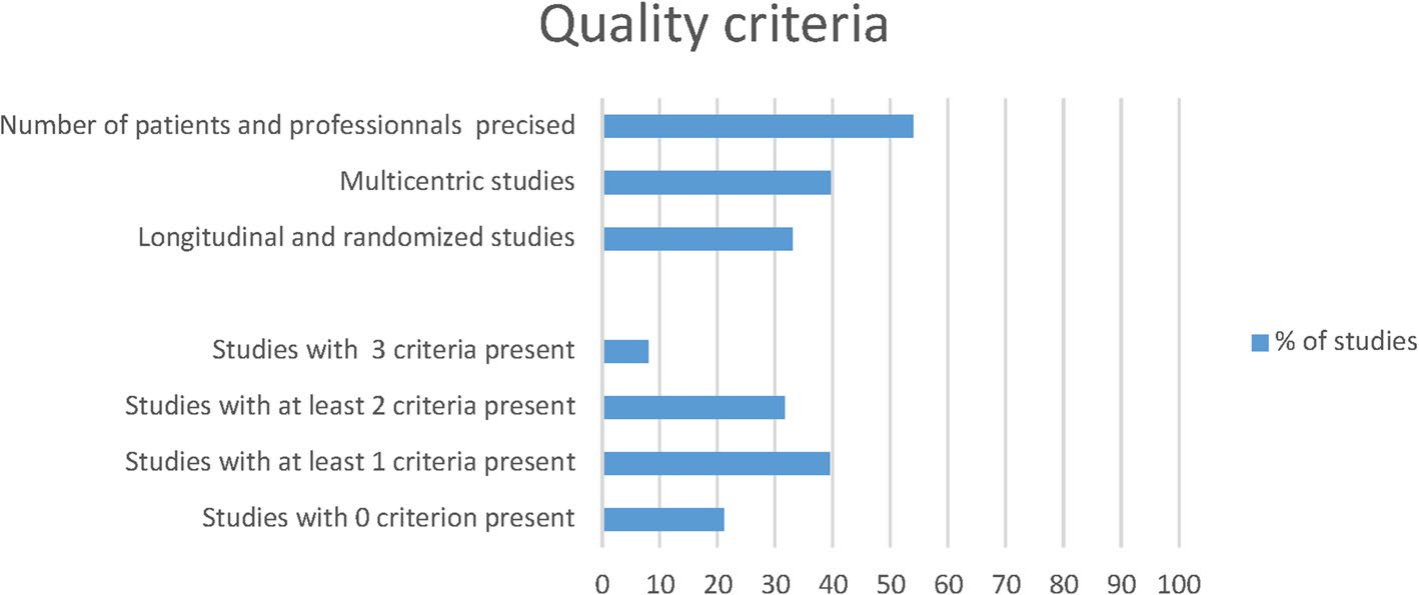
Graphical representation of main methodological items of available studies

Methodological quality of studies is graphically presented in Fig. 3. Of the three criteria used to compose the quality score, 53.9% (*n* = 41) of publications included data on the number of patients and professionals, 39.5% (*n* = 30) were multicenter studies, and 32.9% employed longitudinal or randomized designs (*n* = 25). Overall, 31.57% (*n* = 24), and 7.89% (*n* = 6) of the included studies met respectively 2 and 3 of those criteria. Table 2 represents the number of studies and quality scores by organizational parameters and outcomes. Team composition (*n* = 34) was the most extensively studied determinant with the highest mean quality score (QS = 1.70 [1–3]). Surgical safety (*n* = 53, QS = 1.41 [0–3]) was investigated with better quality score compared to operative time (*n* = 60, QS = 1.21 [0–3].

**Table 2:**
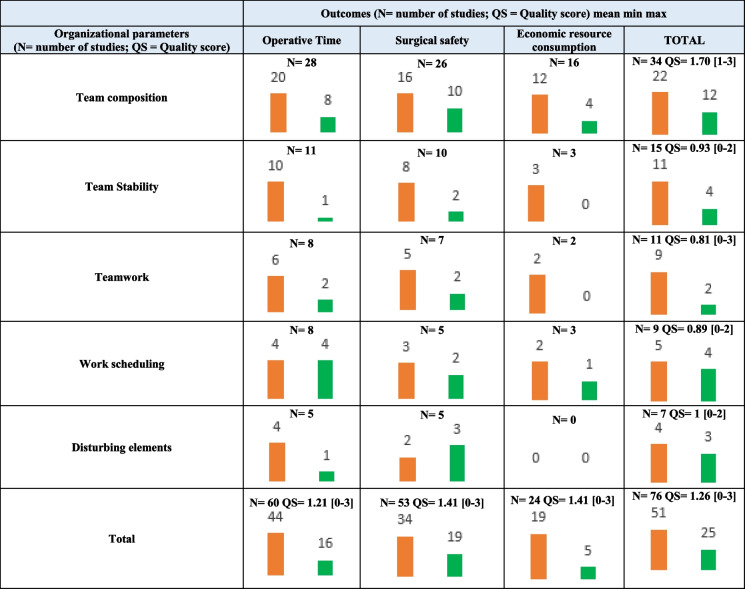
Number and quality score of studies evaluating the effect of organizational parameters on outcomes, according to study design

Study designs were presented in orange for observation cross sectional and intervention quasi experimental designs; observation longitudinal and intervention experimental designs were presented in green

Definitions of organizational factors:

Team composition = number and experience of surgeon, residents, anesthetists, nurses; surgical team relations (work under supervision, involvement of residents, surgeon/resident-nurse-anesthetists relations

Team stability = number of former collaborations (surgeon/resident, surgeon/anesthetist, surgeon/nurse), turnover of surgical and anesthetic team between procedure or during a same procedure

Team work = measuring scales of teamwork, leadership, communication inside team including communication failure

Disturbing elements = number of disturbing elements, type of disturbance, duration of disturbance

Work scheduling = order of scheduling, modifications in scheduling, dedicated operating room, patient turn over, work overlay

*N* number of studies

*QS *mean quality score between 0 to 3 [Range]

**Fig. 4:**
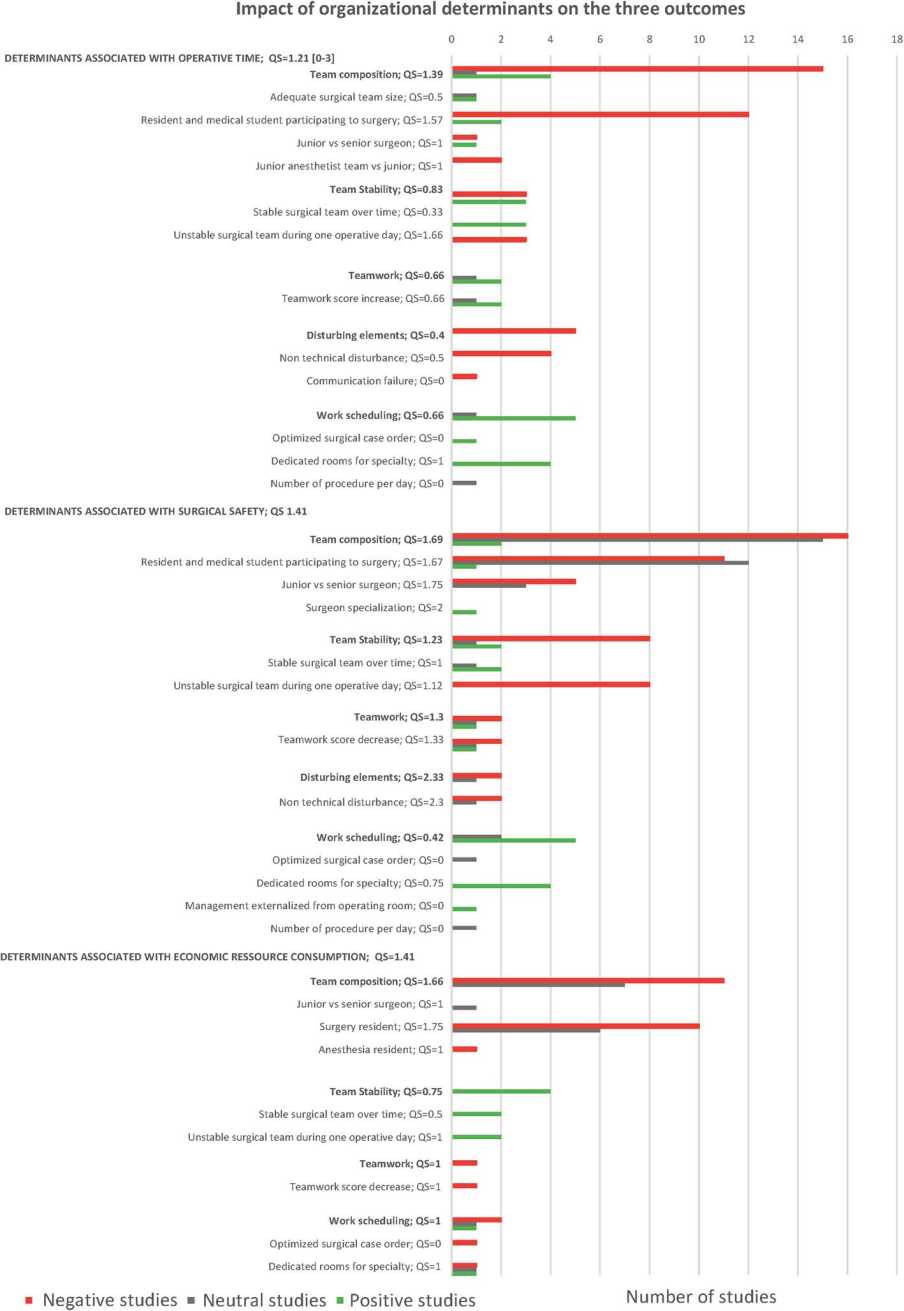
Impact of organizational determinants on outcomes

Additional files 3, 4 and 5: Appendix 3, 4 and 5 provide detailed findings of the selected studies according to each investigated outcome. Corresponding results were summarized in Fig. 4 and hereunder per determinant category.

Surgical team composition was the most extensively studied determinant. On the one hand, having experienced surgeons in the team decreased both the operating time and morbidity rates. Having an experienced anesthesia team also reduced induction times. On the other hand, involving residents during the procedure could led to a longer operating time. Resident participation appeared to result in higher complication rates, redo surgeries, re-hospitalization, length of stay and costs.

Stable surgical teams could reduce both operating time, costs and postoperative morbidity, contrary to changing teams. Five studies found a reduction in complications with stable teams, while team turnover increased the risk of redo surgery and length of stay.

Enhancing teamwork among surgical teams can reduce operative time, as well as implementing standardized collaboration procedures. Conversely, poor teamwork quality was associated with higher postoperative morbidity.

Disturbing elements during surgery were potentially associated with longer operating time and redo surgery.

Regarding work scheduling, the use of specialty-dedicated operating rooms was associated with reduced morbidity and operative time, more patients treated, and saved costs. Appropriate work scheduling was also found to have a positive influence on patient outcomes.

## Discussion

We analyzed the influence of various organizational determinants on surgical performance. Out of the 76 publications that met our inclusion criteria, we found that operating with a specialized [23–32], stable and dedicated surgical team [5, 8, 27, 33–43], and optimizing the operating schedule [44–46], in a room dedicated to the specialty [47–53], leads to improved outcomes. The optimization of teamwork [34, 44, 54–59], as quantified using scales such as OTAS (observational teamwork assessment for surgery), NOTSS (non-technical skills for surgeons), and SPLINTS (scrub practitioners’ list of intraoperative non-technical skills), was found to potentially reduce operative time without affecting the complication rate. Poor teamwork [55, 57], on the other hand, could increase the cost of care [55]. In addition, optimizing teamwork was positively correlated with a decrease in inter-individual communication failures [55, 60]. Scheduling errors or unplanned changes were found to result in a trend to increase operative times [28]. However, optimizing patient scheduling did not influence the complication rate or the number of procedures performed per day [45, 46]. There was mixed evidence regarding the surgical resident involvement: most of studies reported either no association [4, 23, 26, 29, 61–73] or a negative [4, 23, 25, 26, 63–68, 74–84] influence regarding operative time and surgical safety, whereas few found a positive association [28, 30, 76]. Disturbing elements [57, 85–92] and communication failures [93] within the surgical team were found to increase both the operative time and morbidity-mortality rate. The anesthesia team was found to be more frequently affected by disruptions, leading to longer intervals between interventions [88]. One study [89] reported a positive relationship between ambient noise intensity and the rate of general complications.

The objective of this systematic review was to highlight the evidence available in the literature regarding the influence of organizational determinants on operative performance. Our results indicate that available literature is relatively scarce and of poor quality. A preliminary search of PubMed and Scopus showed that thirteen reviews and meta-analyses [6–17, 94] evaluating the influence of organizational factors on surgical performance have been published to date. Most of reported studies in these reviews were not analytical, had few quantitative data, and focused on only one procedure, domain or a limited number of outcomes. An analysis of the relationship between organizational factors and postoperative outcomes or surgical performance was identified in five reviews [6–9, 94]. One review dealt with the evaluation of two scales (OTAS and NOTECHS), and included 14 studies that quantify teamwork, but with no correlation with clinical outcomes [6]. A second review evaluated the impact of intraoperative failure on major complication rate and on hospital mortality. Miscommunication induced 22% of failure during surgery, while equipment failure induced 5.2% of errors [7]. Effective communication is crucial in various stages of surgical procedures, particularly during team turnover. In response to this, Nasiri et al. introduced a handover checklist, resulting in a notable decrease in information omission and an improvement in overall handover quality for scrubs. Although the checklist increased handover duration, it significantly enhanced overall satisfaction, emphasizing its positive influence on communication quality and team contentment within the surgical team [95]. Team familiarity could also improve post operative outcomes according to Awtry et al. who conclude that higher surgeon-anesthesiologist familiarity in cardiac surgery teams correlated with lower rates of adverse outcomes, including 30-day mortality, 90-day mortality, composite morbidity, and the combined endpoint of 30-day mortality or composite morbidity [96]. Two reviews investigated the improvement of team communication on morbidity. Those reviews concluded that pediatric mortality decreased from 2.7% to 1%, and that global mortality decreased from 20.2% to 11% in general surgery after team training for communication [8, 9]. Three reviews evaluated the influence of the participation of residents operating under supervision in simple or complex procedures according to their experience [10, 11, 94]. One of the reviews dealt with team composition^11^ in general without measurement of complications. This study focused on flow disruptions and found a 32.5% delay rate in surgical procedure time. Moreover, team stability led to 24% faster surgery. On the other hand, Bougie et al. [11] evaluated only the bleeding rate and the operative time, which increased. Another review [12] specified the importance of the seniority/experience of the operator on the speed of execution of the procedures and the reduction of unexpected intraoperative events. Some authors also evaluated the impact of the surgeon gender, finding in a study involving 1,165,711 patients, that those treated by female surgeons exhibited lower rates of adverse postoperative outcomes, including mortality at 90 days and 1 year, compared to patients treated by male surgeons, highlighting potential differences in patient outcomes based on physician gender [97].Three reviews have described an effect of music in the operating room, but only evaluated the effects on expert surgeons or surgeons in training who were working on experimental models, rather than in-vivo. The results showed that soft and soothing melodies would promote concentration as opposed to aggressive sounds [13–15]. These studies are similar to the concept of task interruption described in another review [16] that focused on unexpected events (phone calls, cancellations) and their potential per operative influence, but the impacts were not quantified. Other reviews [17] described the influence of team learning and how to use a new tool (the surgical robot) on organization and delays in the operating room. In a study involving robotic prostatectomy, post-intervention console time significantly decreased, dual instrument inactivity was reduced, and the use of dual consoles increased, suggesting that standardizing intraoperative tasks improves efficiency and may enhance operating room capacity [98]. Conversely, simulation-based training across professions showed uniform increases in self-efficacy and motivation, emphasizing the importance of profession-specific and multiprofessional team training [99]. This team training could facilitate access to the operating room and reduce unforeseen events and financial losses due to cancellations. In a study about 933 elective procedures, a high cancellation rate was observed primarily due to a lack of operating room time and inadequate patient preparation, emphasizing the need for improved patient evaluation workflows, sufficient operating room staffing, and punctual start times to enhance operating room efficiency in settings with a high unmet burden of surgical disease [100]. Team learning, involving 40 operating room staff, identified key themes such as a commitment to learning, the significance of a safe space in debriefing, and the role of leadership in mitigating hierarchies [101]. It highlighted the importance of organizational parameters during each surgical step, evolving according to the incoming sequence: beginning, per procedural, and after surgery. Consistent with observations in six surgical departments by Arad et al., machine learning identified 24 contributing factors from each surgical, anesthetic, or circulating nurse work, with varying impacts on wrong site surgeries and retained foreign items, indicating the need for adjusting safety standards based on surgery characteristics and risk assessment in each operating room [102]. The implementation of optimization measures for all these determinants would improve outcomes [103]. Incorporating cognitive support systems (CSTs) in surgical procedures, as indicated by a comprehensive analysis of 37 studies, could result in superior surgical performance compared to traditional methods, manifesting in reduced error rates, enhanced efficiency, and the majority of CSTs exhibiting over 90% accuracy in identifying anatomical markers with an error margin below 5 mm; however, the constrained ergonomic design of current CSTs has impeded broad clinical adoption, underscoring the necessity for additional patient-centered clinical data before the universal integration of CSTs [104]. These studies emphasize the importance of organizational parameters during each surgical step, which evolves according to the incoming sequence: beginning, per procedural, and after surgery [105].

### Limitations of the study

This systematic review was based on 76 quantitative studies that investigated the influence of organizational factors on surgical performance. The data was collected from patients' electronic health records in most studies and covered a wide range of surgical procedures, with digestive and orthopedic surgery being the most represented. Despite the retrospective nature of these publications, the impact was minimized because of quality score assessment. The selection bias was also minimized through a double-blinded review process. The study focused on two databases (PubMed and Scopus), and only included English publications, which limited the scope of the research, and possibly limited the number of determinants that are presented and discussed in this manuscript and may bias to english speaking country outcomes. The majority of the studies (60/76) evaluated the influence of these determinants (team composition, team stability, teamwork, work scheduling, disturbing elements) on operative time, 53/76 on surgical safety and 24/76 on economic resource consumption. OSF registries and institutional databases were not included in the search. It should be noted that there is a lack of studies examining the impact of each determinant individually on each outcome. Specifically, there is limited research on the relationship between team stability and economic resource consumption, teamwork and surgical safety/economic resource consumption, work scheduling and surgical safety/economic resource consumption, as well as disturbing elements and operative time/surgical safety/economic resource consumption. This scarcity of studies represents a limitation in our understanding of the specific associations. Additionally, the majority of the studies, 67 retrospective and 9 prospective, presented low-level evidence. To comprehensively address the diverse and multifaceted nature of our subject, which encompasses various research objectives and methodologies including observational and interventional studies, we opted for a systematic review instead of a meta-analysis. This choice was driven by the challenge of conducting an all-encompassing assessment of methodological quality due to the varied nature of the studies. Our assessment of quality focused on a limited set of three criteria, resulting in a mean quality score. We opted for this limited scale of evaluation instead of validated GRADE evaluation because of the overall poor methodology/heterogeneity in the majority of included studies. When the quality score was 0, we chose to retain the publication in the analysis. The objective was to describe comprehensively the impact of organizational factors, and these studies provide informative elements that allow us to identify trends for further consideration. This enabled us to keep a wide overview of the subject area.

The limited quantity of studies and their substantial heterogeneity prevented a definitive determination of the positive or negative impact of each determinant on outcomes. As a result, the results were presented in terms of median odds ratios and statistically tested values, but many of the data only allow for limited conclusions to be drawn as studies did not provide statistical comparisons. The assessed literature is relatively poor in nature and limits conclusions; on the other hand, this enabled us to throw into relief opportunities for future research.

### Identified gaps and opportunities for future research

The relationship between organizational factors and operative performance is a relatively new field of study. On the other hand, some studies report the analysis of a link between determinants and outcomes that are not quantitatively described, making it impossible to reach a conclusion of statistical association. Most of the research on this topic has been conducted since 2010, and leaves many questions unanswered. The recent growth in data on organizational factors can be attributed to the increased availability of data. The majority of the available data focuses on the influence of the determinants on surgery duration. Team composition and team stability were the two most studied determinants affecting morbidity and mortality. Although various studies have mentioned the relationship between each of the five determinants and economic resource consumption, this outcome still lacks comprehensive investigation and is characterized by a poor quality score. It is currently impossible to determine whether there is a significant association between the other determinants, such as teamwork, work scheduling, and disturbing elements, and operative performance due to the limited number of available studies. Additionally, the clinical relevance of the results has not been clearly established. Many studies only focus on one or two outcomes. These studies do not adjust measured performance according to patient-related factors and the expected complexity/risk of surgery, making it difficult to have a general view of the subject.

The literature on team composition, resident involvement, and their link with operative time and outcomes is abundant but heterogeneous. Some studies report a positive association between increased postoperative morbidity and resident participation, while others report a negative association. However, the results are consistent in showing an increase in operative time related to the participation of residents as surgeons in training.

To further understand the impact of teamwork, it would be necessary to quantify teamwork and assess the association between teamwork and outcomes on validated scales. To date, only 11 studies have been found on this topic, with only 3 of them reporting validated scales correlated with outcomes. The scarcity of studies on teamwork is due to the challenge of analyzing teamwork through declaration of each professional, video analysis, or scales quantifying teamwork in a simple and reproducible manner. From a methodological standpoint, future studies need to improve their quality and level of evidence. The average score we used in this systematic review was relatively low (1.26/3), with a substantial number of retrospective studies. The least studied organizational factors, such as work scheduling and disturbing elements, require further investigation. At this time, there is a lack of data to determine their significant clinical impact. It is essential to conduct new prospective studies to assess the impact of these under-researched organizational factors.

## Conclusion

Recent studies have highlighted the importance of organizational factors in surgical outcomes, particularly the positive impact of specialized and stable team compositions. However, the current literature lacks prospective studies investigating other organizational factors in the operating room environment. Therefore, further prospective quantitative research is needed to enhance our understanding of the broader range of organizational drivers that contribute to surgical performance.

### Supplementary Information

**Additional file 1: Appendix 1.** Search strategy [106, 107].

**Additional file 2: Appendix 2.** Data extraction instrument.

**Additional file 3: Appendix 3.** Determinants associated with operative time [108].

**Additional file 4: Appendix 4.** Determinants associated with surgical safety.

**Additional file 5: Appendix 5.** Determinants associated with economic resource consumption.

## Data Availability

No datasets were generated or analysed during the current study.

## Abbreviations

OTAS: Teamwork assessment for surgery
NOTSS: Non technical skills for surgeons
SPLINTS: Scrub practitioners’ list of intraoperative non technical skills
OSF: Open Science Framework
NOTECHS: Non technical skills
CST: Cognitive support systems
