# Supplementary material for: Operating room organization and surgical performance: a systematic review
Source: Patient Saf Surg. 2024 Jan 29;18:5. doi: 10.1186/s13037-023-00388-3 (PMC10826254; doi:10.1186/s13037-023-00388-3)
Supplement: Supplementary file 1 — Additional file 1: Appendix 1. Search strategy [106, 107]. [file 13037_2023_388_MOESM1_ESM.docx]

**Appendix 1 Search strategy**

### Database: PUBMED

(("Operating Room*"[TIAB] OR "operating rooms"[Mesh] OR "surgical team"[TIAB])

**AND**

(outcome[TIAB] OR complication[TIAB] OR "Adverse event*" [TIAB] OR Mortality [TIAB] OR Death [TIAB] OR Morbidity [TIAB] OR Morbidities [TIAB] OR "Operative time*" [TIAB] OR "Surgical time*" [TIAB] OR performance*[TIAB] OR "operative time"[TIAB] OR "operative efficiency"[TIAB] OR "Patient safety" [TIAB] OR "adverse event*"[TIAB] OR "Patient Outcome Assessment"[Mesh] OR "Postoperative Complications"[Mesh] OR "Intraoperative Complications" [Mesh] OR "Medical errors" [Mesh] OR "Accidental Injuries"[Mesh] OR "operative time"[Mesh] OR "patient safety"[Mesh])

**AND**

(organization*[TIAB] OR "team composition"[TIAB] OR collaboration*[TIAB] OR teamwork*[TIAB] OR "schedule change"[TIAB] OR teaming[TIAB] OR workload*[TIAB] OR "Workload"[Mesh] OR "Appointments and Schedules"[Mesh] OR "Personnel Turnover"[Mesh] OR "Medical Staff, Hospital"[Mesh] OR "Workforce"[Mesh] OR "Personnel Staffing and Scheduling"[Mesh] OR "Personnel Management"[Mesh] OR "Interprofessional Relations"[Mesh] OR "outcome and process assessment"[Mesh] OR "internship and residency"[Mesh] OR "Operating Rooms/organization and administration"[MAJR])

**AND** (English[lang])

**NOT** (Comment[PT] OR Editorial[PT] OR Letter[PT] OR News[PT]))

**Database: SCOPUS**

(TITLE-ABS (Operating Room* OR operating rooms OR surgical team))

**AND**

(TITLE-ABS (outcome OR complication OR Adverse event* OR Mortality OR Death OR Morbidity OR Morbidities OR Operative time* OR Surgical time* OR performance* OR operative time OR operative efficiency OR Patient safety OR adverse event* OR Patient Outcome Assessment OR Postoperative Complications OR Intraoperative Complications OR Medical errors OR Accidental Injuries OR operative time OR patient safety))

**AND**

(TITLE-ABS (organization* OR team composition OR collaboration* OR teamwork* OR schedule change OR teaming OR workload* OR Workload OR Appointments and Schedules OR Personnel Turnover OR Medical Staff, Hospital OR Workforce OR Personnel Staffing and Scheduling OR Personnel Management OR Interprofessional Relations OR outcome and process assessment OR internship and residency OR Operating Rooms/organization and administration))

**AND** (LIMIT-TO ( DOCTYPE,"ar" ) )

**AND** (LIMIT-TO ( LANGUAGE,"English" ) )
