# Supplementary material for: Operating room organization and surgical performance: a systematic review
Source: Patient Saf Surg. 2024 Jan 29;18:5. doi: 10.1186/s13037-023-00388-3 (PMC10826254; doi:10.1186/s13037-023-00388-3)
Supplement: Supplementary file 2 — Additional file 2: Appendix 2. Data extraction instrument. [file 13037_2023_388_MOESM2_ESM.docx]

**Appendix 2 Data extraction instrument**

Data extraction form: Influence of Organizational factors on surgical performance - Systematic Review

| Article number Authors  Date  Journal | |
| --- | --- |
| **Study Design** | |
| Interventional (Randomized controlled trial, Quasi-experimental study) |  |
| - Experimental ( Randomized: individual or cluster randomization) |  |
| - Quasi-experimental (non randomized: before-after without contemporary control group, before-after with contemporary control group, 2 non randomized arms (intervention vs. contemporary control group) without before-after; other) |  |
| Observational study |  |
| - Descriptive or analytical |  |
| - Cross sectional or longitudinal (cohort, case control) |  |
| Precisions (e.g. pre-post study, cluster randomized) |  |
| Prospective / retrospective study |  |
| Origin of data : ad-hoc collection or routinely available |  |
| **Statistics** |  |
| Statistical methods used |  |
| Univariable, bivariable, or multivariable analysis? |  |
| Were confounding factors taken into account? |  |
| **Population and setting** | |
| Geographical area: country, city |  |
| Period |  |
| Mono or multicentric study |  |
| Number of centers |  |
| Status of center(s) |  |
| Surgical specialty |  |
| Adult or pediatric surgery |  |
| Emergency/scheduled procedure |  |
| Surgical procedures: specific procedures or all procedures of the surgical specialty |  |
| Type of surgery: open, laparoscopic/minimally invasive, robotic |  |
| Context (civilian, war, humanitarian surgery) |  |
| Team size |  |
| - Surgeons |  |
| - Inclusion criteria of the surgeon |  |
| - Number of surgeons |  |
| - Attending surgeons |  |
| - Inclusion criteria of the attending surgeon |  |
| - Number of attending surgeons |  |
| - Residents |  |
| - Inclusion criteria of the resident |  |
| - Number of resident |  |
| - Anesthesist |  |
| - Inclusion criteria of the anesthesist |  |
| - Number of anesthesist |  |
| - Nurses |  |
| - Inclusion criteria of the nurses |  |
| - Type of nurses (anesthesiologist, surgical, surrounding) |  |
| Patients |  |
| - Number of patients in the study |  |
| - Number of patients or surgical procedures a day/ a room |  |
| - Inclusion criteria for patients |  |
| - Exclusion criteria for patients |  |
| **Objective** | |
| Main objective of the study |  |
| Secondary objectives (scheduling, workload, teamwork, disturbing elements, other) |  |
| Is the study of the organizational parameters the main or a secondary objective of the study? |  |
| Are the organizational parameters considered as a determinant or as an outcome? |  |
| **Organizational parameters influencing surgical performance** | |
| Surgical team in the operating room |  |
| - Number of surgeons during each procedure |  |
| - Level of experience of surgeon (how many years of practice, age, level of expertise, etc.) |  |
| - Number of attending surgeons during each procedures |  |
| - Level of experience of attending surgeon (how many years of practice, age, level of expertise, etc.) |  |
| - Number of resident during each procedure |  |
| - Level of experience of resident (how many years of practice, age, level of expertise, etc.) |  |
| - Number of anesthesist during each procedure |  |
| - Level of experience of anesthesist (how many years of practice, age, level of expertise, etc.) |  |
| - Number of nurses for each subtype |  |
| - Level of experience of nurse (how many years of practice, age, level of expertise, etc.) |  |
| - Changing surgical team during procedure |  |
| - Consistency of team/ turn over between procedures |  |
| - Turnover of circulating nurse |  |
| - Turnover of instrumenting nurse |  |
| - Turnover of anesthetic team |  |
| - Total number of people in the OR |  |
| - Procedure performed by resident under supervision |  |
| Team work behavior/surgical team relations/safety climate |  |
| - Measuring scale used yes/no |  |
| - - Team Familiarity |  |
| - - Unprofessional behavior |  |
| - - Safety climate |  |
| - - Team work behavior |  |
| - - Team performance |  |
| - - Team interactions |  |
| - Number of former collaborations between each team members |  |
| - - Surgeon/resident |  |
| - - Surgeon/anesthesiologist |  |
| - - Surgeon/nurse |  |
| - - Communication failure.success |  |
| - Surgical team relations (name and scale values) |  |
| - - Surgeon/resident relation |  |
| - - Surgeon/nurse relation |  |
| - - Surgeon/anesthesiologist relation |  |
| - Resident participation rate during procedure |  |
| Disturbing elements |  |
| - Total number of disturbing elements |  |
| - Type of disturbing element / task interruption |  |
| - Number of disturbing elements / task interruptions |  |
| - Duration of disturbance / task interruption |  |
| Scheduling / workload |  |
| - Order of scheduling |  |
| - Modifications in scheduling |  |
| - Dedicated operating room |  |
| - Turn over |  |
| - Work overlay |  |
| Other factor (Temperature / Music…) |  |
| **Outcomes** of the **Organizational parameters** | |
| Per procedural datas |  |
| - Anesthesia time (duration) |  |
| - Operative time (duration) |  |
| Team work behavior |  |
| - Quantification on scale |  |
| Number of procedures a day |  |
| Turn over of patients |  |
| Team turn over |  |
| Communication failure / success |  |
| Per operative accident/error |  |
| - Bleeding |  |
| - Quantification of bleeding |  |
| - Change in procedure / conversion etc. |  |
| Post-operative datas |  |
| - Patients' morbidity measurement (yes/no) |  |
| - Patients' mortality measurement (yes/no) |  |
| - Delay of morbidity measurement (yes/no) |  |
| - Delay of morbidity |  |
| - Delay of mortality measurement (yes/no) |  |
| - Delay of mortality |  |
| - DINDO Clavien classification (yes/no) |  |
| - DINDO Clavien classification grade |  |
| Type of post-operative complications |  |
| - Bleeding |  |
| - Quantification of bleeding |  |
| - General morbidity |  |
| - Specific morbidity |  |
| - Surgical site infection |  |
| Return to the operating room |  |
| Transfer to intensive care unit |  |
| Length of stay |  |
| Readmission |  |
| Economic impact |  |
| - Cost effectiveness |  |
| - Theatre operating cost |  |
| **Main findings** | |
| Reported results corresponding to the main objective of the authors |  |
| Reported results of interest for our review |  |
