# Supplementary material for: Operating room organization and surgical performance: a systematic review
Source: Patient Saf Surg. 2024 Jan 29;18:5. doi: 10.1186/s13037-023-00388-3 (PMC10826254; doi:10.1186/s13037-023-00388-3)
Supplement: Supplementary file 3 — Additional file 3: Appendix 3. Determinants associated with operative time [108]. [file 13037_2023_388_MOESM3_ESM.docx]

**Appendix 3: Determinants associated with operative time.**

| **Determinant** | **Operative time** | **Mean quality score of studies [min-max]** |
| --- | --- | --- |
| **Team composition n=28** |  |  |
| **Adequate surgical team size n=2** |  | 0.5 [0-1] |
| 1 Positive study ^43^ | - 67 min | 1 |
| 1 Negative study ^106^ | +4.8 min | 0 |
| 0 Neutral study | - | - |
| **Resident and medical student**  **participating to surgery n=14** |  | 1.57 [1-3] |
| 2 Positive studies ^28, 30^ | -15.19 min | 2 [2-] |
| 12 Negative studies ^75,76, 25, 77, 63, 64, 78,79, 65, 80, 81, 68^ | + 20.5 min (-15.19 - +87) | 1.5 [1-3] |
| 0 Neutral study | - | - |
| **Junior vs senior surgeon n=2** |  | 1 [1] |
| 1 Positive study ^32^ | -70 min | 1 [1] |
| 1 Negative study ^60^ | + 30 min | 1 [1] |
| 0 Neutral study | - | - |
| **Junior anesthetist team vs senior n=2** |  | 1 [1] |
| 0 Positive study | - | - |
| 2 Negative studies ^60, 32^ | + 33.69 min (5.1-62.28) | 1 [1] |
| 0 Neutral study | - | - |
| **Team stability n=11** |  |  |
| **Stable surgical team over time n=3** |  | 0.33 [0-1] |
| 3 Positive studies ^33, 39 107^ | -47.1 min  24% reduction  -38.5min | 0.33 [0-1] |
| 0 Negative study | - | - |
| 0 Neutral study | - | - |
| **Unstable surgical team during one operative day n=3** |  | 1.66 [1-3] |
| 0 Positive study | - | - |
| 3 Negative studies ^5, 36, 40,^ | OR 1.52 (1.20-1.91)  + 44.2 min (34.7-53.7) | 1.66 [1-3] |
| 0 Neutral study | - | - |
| **Teamwork n=8** |  |  |
| **Teamwork score increase n=3** |  | 0.66 [0-2] |
| 2 Positive studies ^31, 32^ | 24% decrease  -10 min | 1 [0-2] |
| 0 Negative study | - | - |
| 1 Neutral study ^27^ | No impact | 0 [0] |
| **Disturbing elements n=5** |  |  |
| **Non-technical disturbance n=4** |  | 0.5 [0-1] |
| 0 Positive study | - | - |
| 4 Negative studies ^85, 86, 87, 90^ | + 21 min (10-31)  +3 min 36 sec per disturbance | 0.5 [0-1] |
| 0 Neutral study | - | - |
| **Communication failure n=1** |  | 0 [0] |
| 0 Positive study | - | - |
| 1 Negative study ^88^ | Longer room in to room out | 0 [0] |
| 0 Neutral study | - | - |
| **Work Scheduling n=8** |  |  |
| **Surgical case order n=1** |  | 0 [0] |
| 1 Positive study ^46^ | Later in the day -33 min | 0 [0] |
| 0 Negative study | - | - |
| 0 Neutral study | - | - |
| **Dedicated rooms for specialty n=4** |  | 1 [0-2] |
| 4 Positive studies ^47, 48, 49, 51^ | -23.85 %  -19.76 min (-49.3 -3) | 1 [0-2] |
| 0 Negative study | - | - |
| 0 Neutral study | - | - |
| **Number of procedure per day n=1** |  | 0 [0] |
| 0 Positive study | - | - |
| 0 Negative study | - | - |
| 1 Neutral study ^45^ | R −9.65; 95% CI (−29.26 to 9.94); p = 0.334 | 0 [0] |

**Data expressed according to available information in manuscripts: Odds ratio with 95% CI or median (min-max) when several studies concluded with a quantified time observation. When statistical analysis was presented and multiple results found, median value was calculated. Significant results enabled to classify between positive and negative studies. When statistical analysis was not significant or not performed, results were classified in the neutral section.**

**The average quality score of the studies presented for each outcome is presented on a scale of 0 to 3 for each quantitative value reported**
