# Supplementary material for: Operating room organization and surgical performance: a systematic review
Source: Patient Saf Surg. 2024 Jan 29;18:5. doi: 10.1186/s13037-023-00388-3 (PMC10826254; doi:10.1186/s13037-023-00388-3)
Supplement: Supplementary file 4 — Additional file 4: Appendix 4. Determinants associated with surgical safety. [file 13037_2023_388_MOESM4_ESM.docx]

**Appendix 4: Determinants associated with surgical safety**

| **Determinant** | **Post-operative complications** | | | | **Mean quality score of studies [min-max]** |
| --- | --- | --- | --- | --- | --- |
|  | **Overall morbidity** | **Overall mortality** | **Redo surgery** | **Readmission** |  |
| **Team composition n=26** |  |  |  |  |  |
| **Resident and medical student**  **participating to surgery n=18** |  |  |  |  | 1.67 [1-3] |
| 1 Positive study ^76^ | OR median 0.5 (0.16-0.93) | - | - | - | 1 [1] |
| 11 Negative studies ^4, 74, 75, 76, 63, 78, 65, 80, 82, 67, 32^ | OR median 1.33 (1.09-9.05)  +2.2% (0.7-9.1) | + 0.2% | OR median 1.25 (1.09-1.93)  +0.6% | +2.4% p=0.008  OR 1.55 (1.17-2.06) | 1.54 [1-2] |
| 12 Neutral studies ^23, 4, 61, 62, 26, 63, 64, 65, 29, 66, 67, 68^ | OR 1.00 (0.5-1.55)  No impact | OR median 1.18 (0.6-1.52)  No impact | OR median 1.34 (0.9-1.7)  +0.2% (0.1-0.4)  No impact | OR median 1.05 (0.83-1.4)  No impact | 1.83 [1-3] |
| **Junior vs senior surgeon n=8** |  |  |  |  | 1.75 [1-3] |
| 0 Positive study | - | - | - | - | - |
| 5 Negative studies ^23, 25, 26, 27, 29^ | + 0.55% (0.13-1.6) | - | +0.9% (0.2-1.6) | +1.2% p=0.24 | 1.2 [1-2] |
| 3 Neutral studies ^79, 65, 66^ | No impact | No impact | - | - | 2.66 [2-3] |
| **Surgeon specialization n=1** |  |  |  |  | 2 [2] |
| 1 Positive study ^31^ | OR 0.54 (0.31-0.95) p=0.034 | OR 0.51 (0.30-0.86) p=0.011 | OR, 0.58; 95% CI, 0.35-0.97; P= .038) | - | 2 [2] |
| 0 Negative study | - | - | - | - | - |
| 0 Neutral study | - | - | - | - | - |
| **Team stability n=10** |  |  |  |  |  |
| **Stable surgical team over time n=3** |  |  |  |  | 1 [0-2] |
| 2 Positive studies ^40, 43^ | - 6.32% (-0.25 to -12.4) | - | - | - | 0.5 [0-1] |
| 0 Negative study | - | - | - | - | - |
| 1 Neutral study ^31^ | No impact | - | - | - | 2 [2] |
| **Unstable surgical team during one operative day n=8** |  |  |  |  | 1.12 [0-2] |
| 0 Positive study | - | - | - | - | - |
| 8 Negative studies ^34, 35, 36, 37, 40, 27, 42, 43^ | + 10% (1.5-19.7)  +52ml blood loss | + 1% p=0.31 | +16.7% p=0.014 | +4% p=0.011  OR 1.42 (1.07-1.89) | 1.12 [0-2] |
| 0 Neutral study | - | - | - | - | - |
| **Teamwork n=7** |  |  |  |  |  |
| **Teamwork score decrease n=4** |  |  |  |  | 1.33 [1-2] |
| 1 Positive study^56^ | Decrease | - | - | - | 1 [1] |
| 2 Negative studies ^55, 57^ | +13 % (11.9-14,1)  Increase | - | - | - | 1.5 [1-2] |
| 1 Neutral study ^56^ | - | +1.2% p=0.24  No impact | - | - | 1 [1] |
| **Disturbing elements n=5** |  |  |  |  |  |
| **Non-technical disturbance n=3** |  |  |  |  | 2.33 [2-3] |
| 0 Positive study (n= 0) | - | - | - | - | - |
| 2 Negative studies ^89, 57^ | LHR 0.042 p<0.05 | - | + 3.5% p=0.87 | - | 2 [2] |
| 1 Neutral study ^60^ | - | No impact | - | - | 3 [3] |
| **Work scheduling n=5** |  |  |  |  |  |
| **Surgical case order n=1** |  |  |  |  | 0 [0] |
| 0 Positive study | - | - | - | - | - |
| 0 Negative study | - | - | - | - | - |
| 1 Neutral study ^46^ | No impact | - | - | - | 0 [0] |
| **Dedicated room n=4** |  |  |  |  | 0.75 [0-2] |
| 4 Positive studies ^47, 48, 49, 50^ | -7.6% (4.7-17) | -5.6% | - | - | 0.75 [0-2] |
| 0 Negative study | - | - | - | - | - |
| 0 Neutral study | - | - | - | - | - |
| **Management externalized from operating room n=1** |  |  |  |  | 0 [0] |
| 1 Positive study ^44^ | Decrease | - | - | - | 0 [0] |
| 0 Negative study | - | - | - | - | - |
| 0 Neutral study | - | - | - | - | - |
| **Number of procedure per day n=1** |  |  |  |  | 0 [0] |
| 0 Positive study | - | - | - | - | - |
| 0 Negative study | - | - | - | - | - |
| 1 Neutral study ^45^ | OR1.08 (0.67-1.73) | OR1.03 (0.12-8.53) | - | - | 0 [0] |

**When statistical analysis was presented and multiple results found, median value was calculated. Significant results enabled to classify between positive and negative studies. When statistical analysis was not significant or not performed, results were classified in the neutral section.**

**The average quality score of the studies presented for each outcome is presented on a scale of 0 to 3 for each quantitative value reported**
