# Supplementary material for: Operating room organization and surgical performance: a systematic review
Source: Patient Saf Surg. 2024 Jan 29;18:5. doi: 10.1186/s13037-023-00388-3 (PMC10826254; doi:10.1186/s13037-023-00388-3)
Supplement: Supplementary file 5 — Additional file 5: Appendix 5. Determinants associated with economic resource consumption. [file 13037_2023_388_MOESM5_ESM.docx]

**Appendix 5: Determinants associated with economic resource consumption**

| **Determinant** | **Economic impact** | | **Mean quality score of studies [min-max]** |
| --- | --- | --- | --- |
|  | **Costs** | **Length of stay** |  |
| **Team composition n=16** |  | |  |
| **Junior vs senior surgeon n=1** |  |  | 1 [1] |
| 0 Positive study | - | - | - |
| 0 Negative study | - | - | - |
| 1 Neutral study ^29^ | - | No difference | 1 [1] |
| **Surgery resident n=16** |  |  | 1.75 [1-3] |
| 0 Positive study | - | - | - |
| 10 Negative studies ^23, 108, 76, 26, 63, 79, 65, 81, 66, 68^ | +3141.95 dollars per year per resident  +661 dollars per patient | odds ratio = 1.53, p < 0.001  +0.55 days (0-1.7) | 1.8 [1-3] |
| 6 Neutral studies ^4, 62, 75, 25, 41, 7^ | - | No impact | 1.67 [1-3] |
| **Anesthesia resident n=1** |  |  | 1 [1] |
| 0 Positive study | - | - | - |
| 1 Negative study ^24^ | +228.73 dollars per patient | - | 1 [1] |
| 0 Neutral study | - | - | - |
| **Team stability n=3** |  | |  |
| **Stable surgical team over time n=2** |  |  | 0.5 [0-1] |
| 2 Positive studies ^40, 43^ | -2093 euros per patient | -2 days p=0.02 | 0.5 [0-1] |
| 0 Negative study | - | - | - |
| 0 Neutral study | - | - | - |
| **Unstable surgical team during one operative day n=2** |  |  | 1 [1] |
| 0 Positive study | - | - | - |
| 2 Negative studies ^36, 41^ | - | OR 1.51 (1.23–1.86)  + 0.3 days (0.2-0.3) | 1 [1] |
| 0 Neutral study | - |  | - |
| **Team work n=2** |  | |  |
| **Teamwork score decrease n=1** |  |  | 1 [1] |
| 0 Positive study | - | - | - |
| 1 Negative study ^55^ | Resource waste p=0.002 | - | 1 [1] |
| 0 Neutral study | - | - | - |
| **Work scheduling n=3** |  | |  |
| **Surgical case order n=1** |  |  | 0 [0] |
| 0 Positive study | - | - | - |
| 1 Negative study ^46^ | - | +22% of delayed stay | 0 [0] |
| 0 Neutral study | - | - | - |
| **Dedicated room n=2** |  |  | 1 [0-2] |
| 1 Positive study ^48^ | +2 patients treated a day | - | 2 [2] |
| 1 Negative study ^48^ | +4.74 dollars per minute | - | 2 [2] |
| 1 Neutral study ^50^ | - | No diff | 0 [0] |

**When statistical analysis was presented and multiple results found, median value was calculated. Significant results enabled to classify between positive and negative studies. When statistical analysis was not significant or not performed, results were classified in the neutral section.**

**The average quality score of the studies presented for each outcome is presented on a scale of 0 to 3 for each quantitative value reported**
